# Prevalence of undiagnosed type 2 diabetes in South Asia: A systematic review and meta-analysis

**DOI:** 10.1007/s12020-026-04711-7

**Published:** 2026-08-01

**Authors:** Faiza Afzal, Imtiaz Ahmad, Kainat Khalid, Ikram Bashir, Sohail Akhtar

**Affiliations:** 1 Statistics department, Government College University (GCU), Lahore, Pakistan; 2 Programa de Pós-Graduação Em Bioquímica e Bioprospecção, Universidade Federal de Pelotas, Pelotas, Brasil; 3 Programa de Pós-Graduação em Enfermagem, Universidade Federal de Santa Maria, Rio Grande do Sul, Santa Maria, Brasil; 4 Laboratório de Botânica, Universidade do Vale do Taquari (Univates), Lajeado, Rio Grande do Sul Brasil; 5 Department of Statistics, Faculty of Science, University of Tabuk, Tabuk, Saudi Arabia

**Keywords:** Type 2 diabetes mellitus, Prevalence, Undiagnosed diabetes, South Asia, Meta-analysis

## Abstract

**Purpose:**

Although South Asia exhibits a considerable burden of type 2 diabetes mellitus (T2DM), the numbers of undiagnosed T2DM remains inadequately characterized at the regional level. This systematic review and meta-analysis aimed to approximate the pooled prevalence of undiagnosed T2DM among the general adult population and to assess country-specific variations in South Asian countries.

**Methods:**

A comprehensive literature search was directed in PubMed, Scopus, Cochrane Library, ScienceDirect, and Web of Science for studies published between 1994 and 2022. Observational studies reporting the prevalence of undiagnosed T2DM among adults aged 20–79 years in South Asia were included. By using logit transformation, Random-effects meta-analysis was conducted to assess pooled prevalence. While using Cochran’s Q and I² statistics Heterogeneity was calculated, publication bias was assessed using funnel plots and Egger’s test, and subgroup analyses were performed by country.

**Results:**

In this review, a total of 28 studies containing 104,019 participants and 5719 undiagnosed T2DM cases were included. The observed pooled prevalence of undiagnosed T2DM was 6.67% (95% CI: 4.99–8.58%) with considerable uncertainty as indicated by a very wide prediction interval (0.33%–19.75%) and extreme heterogeneity (I² = 98.5%, *p* < 0.001). At country-level substantial variation was seen, with the highest prevalence in Sri Lanka (30.54%; 95% CI: 17.90–44.82%), followed by Nepal (7.02%; 95% CI: 4.24–10.44%), Pakistan (5.97%; 95% CI: 3.97–8.33%), Bangladesh (5.84%; 95% CI: 5.28–6.42%) and India (5.26%; 95% CI: 3.66–7.14%). The results of Egger’s test (t = 1.84, *p* = 0.0773) suggested no statistical significance evidence of publication bias.

**Conclusion:**

These findings should be interpreted with attentiveness because of extensive heterogeneity and variability across studies. However, the high number of undiagnosed T2DM in general population of South Asia reflects a great burden and indicates inter-country differences. Therefore, this conclusion highlights the need for advanced surveillance systems, valid diabetes screening, and approach to health care facilities, especially in deprived and high-risk population in the region.

**Supplementary Information:**

The online version contains supplementary material available at 10.1007/s12020-026-04711-7.

## Introduction

Type 2 diabetes mellitus (T2DM) is a non-communicable metabolic disease with persistent high glucose level arising from two pathological features, insulin resistance and progressive pancreatic β-cell disfunction, leading to insufficient insulin secretion [1, 2]. T2DM indicates one of the main worldwide epidemics of the 21st century, as per report of International Diabetes Federation (IDF), in 2021 around 537 million people were living with diabetes, a prediction that may get to 783 million by 2045 [3–5]. The South Asian region (composed of India, Pakistan [6], Bangladesh [7], Nepal, Sri Lanka [8], Bhutan, Maldives, and Afghanistan [9]) position as a major hotspot of the diabetes burden, it is home to about a quarter of the world’s population and already concentrates more than 90 million diagnosed cases [10, 11]. However, this burden is likely to be greatly underrated due to the high number of undiagnosed T2DM, shoved by limited access to screening, delayed diagnosis, and uneven healthcare infrastructure across countries and between rural and urban settings [12, 13].

In the current study, “undiagnosed diabetes” indicates to the number of previously undetected T2DM in the general adult population, rather than the prevalence of undiagnosed numbers among individuals with diabetes. Undiagnosed diabetes is a serious nation health problem because people with asymptomatic hyperglycemia remain exposed for years to micro- and macrovascular complications without remedial intervention or lifestyle change [14–16]. Population studies in India expose number of undiagnosed diabetes ranging from 40% to 70% of total cases, meaningly higher than those observed in European or North American populations [11, 17–19]. Factors such as augmented urbanization, nutritional evolution with bigger consumption of refined carbohydrates and cheap vegetable oils, sedentary lifestyle, genetic predisposition (Indian “thin-fat phenotype” effect), and structural limitations of health systems back to this issue [20, 21].

Numerous cross-sectional studies performed over the past 20 years have employed diagnostic criteria from the World Health Organization (WHO) or the American Diabetes Association (ADA) fasting blood glucose ≥ 126 mg/dL, 2 h post-overload blood glucose ≥ 200 mg/dL, or HbA1c ≥ 6.5% in demonstrative samples from rural and urban areas of South Asia [22–24]. But, heterogeneity in methodology like different screening method, age groups, definition of urban/rural area and also the lack of population based studies make it hard to get an overall estimate of the real number of undiagnosed diabetes in the region [22, 25].

Therefore, the current study aims to conduct a meta-analysis that collects data from published literature, allowing the scheming of a pooled prevalence of undiagnosed T2DM in South Asia, evaluating the effect of main factors like gender, area of residence, diagnostic criteria, period of study and identifying knowledge gaps. The findings of this meta-analysis may support public policies for opportunistic screening, reinforce the need to include HbA1c in national health programs, and guide the allocation of resources to regions of greater vulnerability.

## Materials and methods

### Registration

Protocol for this systematic review and meta-analysis was developed in accordance with the PRISMA guidelines [26]. The protocol was registered in the PROSPERO database with the registration number CRD42025638236, and complete details are available online.

### Study design

This systematic review and meta-analysis examined the prevalence of undiagnosed type 2 diabetes mellitus (T2DM) among the general population in South Asian adults (aged 20–79 years) from 1994 to 2022. All studies used reported crude prevalence estimate. Prevalence was defined as the proportion of individuals with T2DM in the study population and the values were extracted directly from the included studies without recalculated or age standardization. The review followed PRISMA guidelines for transparency and reproducibility.

### Data sources and search strategy

Electronic databases were searched: PubMed, ScienceDirect, Scopus, Cochrane Library, Web of Science, EMBASE, and Google Scholar. Search terms were grouped into three categories: condition (T2DM or related terms), ethnicity (South Asian countries), and outcome (prevalence or epidemiology). Keywords within categories were combined using “OR,” and categories using “AND.” Database-specific strategies are detailed in Table S1 (Supplementary File) Reference lists of included studies were hand-searched using a snowballing approach. Regarding language restrictions, only English-language publications were included due to resource constraints.

### Inclusion and exclusion criteria

Studies were included if they: (1) were observational (cross-sectional or cohort) or population-based surveys reporting T2DM prevalence; (2) focused on South Asian adults (or provided disaggregated data for South Asians in mixed-ethnicity samples); (3) were conducted in South Asian countries or among South Asian populations globally; and (4) reported prevalence for adults aged 20–79 years.

Studies were excluded if they: (1) were reviews, editorials, letters, protocols, case reports, or conference abstracts; (2) involved non-South Asian populations (50% non-South Asian in mixed samples); (3) focused on children, animals, T2DM management, causation, or risk factors without prevalence data; (4) were non-English; or (5) reported only unpublished or interim data.

### Study selection

**Fig. 1:**
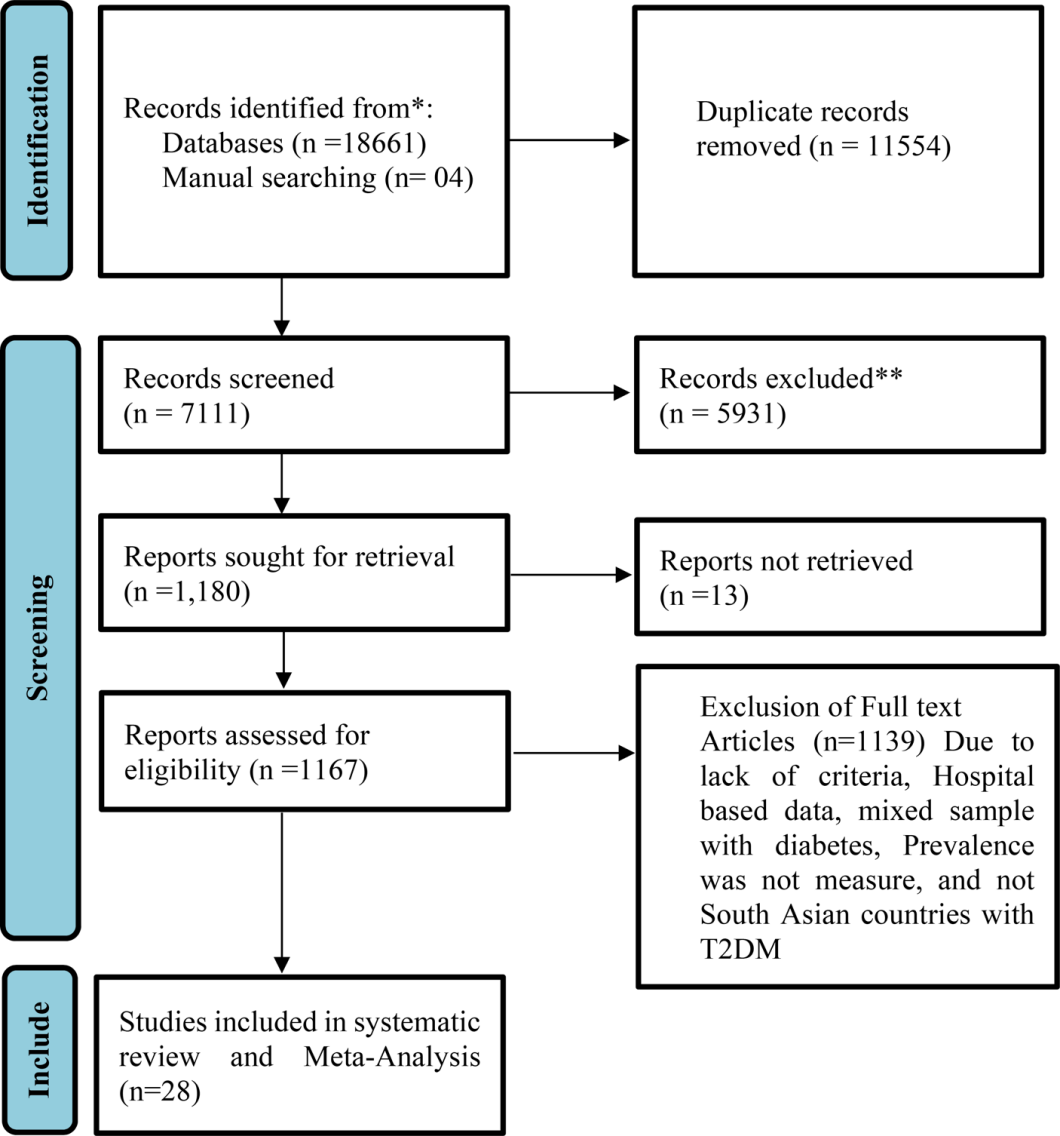
PRISMA flowchart of studies included in this systematic review and meta-analysis

Records from electronic and manual searches were imported into Covidence (www.covidence.org) for deduplication [27]. Two reviewers independently screened titles and abstracts, followed by full-text assessment for eligibility. Disagreements were resolved by consensus or a third reviewer. Reasons for exclusion were documented in Fig. 1.

### Quality assessment

For quality assessment a group of three expert was appointed, they independently assessed the methodological quality by applying the Joanna Briggs Institute (JBI) Critical Appraisal Checklist for Prevalence Studies [28]. Basically, this approach evaluates studies by focusing on major domains, including sample frame appropriateness, sampling method, sample size adequacy, data analysis, and measurement reliability. Each question was ranked as “yes,” “no,” “unclear,” or “not applicable,” rather than producing a summated score [29]. Disagreements were resolved through discussion or negotiation by a third reviewer. Assessments are reported in table S2.

### Data extraction

Data was extracted by one reviewer and verified by supervisors using a standardized data extraction form developed in accordance with the Joanna Briggs Institute (JBI) guidelines. Extracted variables included: study title, country, participant characteristics (age, sex, sample size), recruitment method, prevalence estimates (with 95% CIs), diagnostic criteria, and adjustment methods. For multi-publication studies, data were collated to avoid duplication. Other authors reviewed the extracted data to find the missing data if necessary.

### Data synthesis and statistical analysis

All statistical analyses were done using R V.4.6.0 (RStudio version). Meta-analyses were conducted using two packages: “meta” [30] and “metafor” [31]. We combined the effect estimates assuming Der Simonian-Laird inverse variance random effects model, which were visualized using forest plots [32]. In contrast to fixed effects models, random effects models are more conservative and have good properties under conditions of heterogeneity because random effects models take both within-study and between-study variances into consideration [32]. Freeman-Tukey double arcsine transformation was used as a variance stabilizing procedure before calculation of the pooled estimates [33]. Heterogeneity was estimated by applying the chi-square test on Cochran’s Q statistic, which was calculated based on H and I² indices. The I² index gives the estimate of the percentage of total variation across the studies attributable to true heterogeneity rather than chance. Subgroup analysis was performed to identify the probable causes of heterogeneity. The covariates included were geographical location, year of publication, sample size, year of data collection, gender, methodological quality, and mean age of participants. Symmetry of the funnel plots was assessed along with the Egger’s regression test [34] to identify publication bias, *p* < 0.05 was considered statistically significant. Cohen’s κ [35] was calculated to assess inter-rater reliability between the investigators in selecting studies and extracting data.

## Results

### Study selection and characteristics

The literature search across PubMed, Google Scholar, Web of Science, Scopus, and manual searches of South Asian research journals yielded 18665 records. After removing 11554 duplicates (accounting for overlaps across country-specific searches. 7,111 unique records remained. Then 5,931 irrelevant studies were excluded during title and abstract screening, the rest of studies (1167) were subjected for full-text review. Of these, 1139 were removed due to lack of numerical prevalence data, non-South Asian settings, unclear diagnostic techniques, hospital-based data, or failure to meet eligibility standards. Finally, 28 studies met inclusion criteria: Bangladesh (*n* = 4), India (*n* = 10), Pakistan (*n* = 8), Nepal (*n* = 4), and Sri Lanka (*n* = 2). The PRISMA flow diagram is shown in Fig. 1.

Characteristics of included studies are summarized in Table 1. Studies spanned 1994–2022, with sample sizes ranging from 33 to 19,211 (total *N* = 104019). Participants were adults ≥ 18 years (mean age 20–85 years). Undiagnosed T2DM was defined via fasting plasma glucose ≥ 7.0 mmol/L or HbA1c ≥ 6.5% per WHO criteria, without prior diagnosis. Prevalence of undiagnosed T2DM ranged from 2.18% (India) to 36% (Sri Lanka), with higher rates in urban/semi-urban areas. Comorbidities included hypertension (8.2–65.4%), obesity (11.5–60.7%), and smoking (8.1–27%). Data extraction followed Cochrane EPOC guidelines, prioritizing sensitive assays (e.g., fasting glucose over self-report) and recent criteria (WHO 2006 over 1999). One stratum per study was selected to avoid double-counting, prioritizing the most representative estimate of the general population (e.g., overall sample or largest subgroup).

**Table 1:** Basic characteristics of Included Studies (*n* = 28)

Country	Author	Reference	Data collection year	Sample size	Positives	Participant avg. age (year)	Research Design	Setting	Undiagnosed Diabetes (Prevalence)	Sampling	Hypertension %	Obese %	Smoker %
Bangladesh	Hossain, et al. (2022)	[36]	2017–18	11,952	722	18–95	Survey	Rural & Urban	6.040829987	Two stage sampling random	28.6	17.3	NA
Bangladesh	Sayeed et al. (2004)	[37]	2002	1,480	67	≥ 20	Survey	Rural	4.527027027	Cluster sampling	NA	NA	NA
Bangladesh	Rahim (2007)	[38]	2004	3981	244	≥ 20	Survey	Semi-Urban	6.129113288	Simple random	NA	NA	NA
Bangladesh	Bhowmik et al. (2012)	[39]	2009	2293	141	20–80	Cross-sectional	NA	6.149149586	Cross-sectional	NA	NA	NA
India	Sadikot et al. (2004)	[40]	1999–2002	18,363	581	≥ 25	Survey	National	3.163971029	multi step	NA	NA	NA
India	Menon et al. (2006)	[41]	2002–2005	986	85	18	Cross-sectional	Urban	8.620689655	simple random	NA	NA	NA
India	Chow et al. (2006)	[42]	2005	4535	308	≥ 30	Survey	Rural	6.791620728	Stratified random sample	NA	NA	NA
India	Vijayakumar et al. (2009)	[43]	2007	1645	43	≥ 20	Cross-sectional	Rural	2.613981763	cluster-based census sampling	NA	NA	NA
India	Ravikumar et al. (2010)	[44]	2008–2009	2227	139	≥ 20	Cross-sectional	Urban	6.241580602	Stratified systematic random	NA	NA	NA
India	Zaman et al. (2011)	[45]	2009–2010	1370	79	≥ 20	Cross-sectional	Rural	5.766423358	Simple random	65.13	NA	NA
India	Agarwal, et al. (2017)	[46]	2013–2014	1209	32	≥ 30	Cross-sectional	Rural	2.64681555	Simple Random	49.4	13.3	NA
India	Arora, et al. (2010)	[47]	2007–2008	1003	55	18	Cross-sectional	Urban	5.483549352	Cross-sectional	NA	NA	NA
India	Dasappa, et al. (2015)	[48]	2012–2013	2013	233	59	Cross-sectional	Urban	11.57476403	census-type sampling	44.9	11.9	8.1
India	Deo, et al. (2006)	[49]	2006	1022	29	20	Cross-sectional	Rural	2.837573386	Multistage cluster	NA	NA	NA
Pakistan	Aamir, et al. (2018)	[50]	2017	18,856	1,577	≥ 20	Survey	Rural & Urban	8.36338566	Multistage stratified cluster	NA	NA	NA
Pakistan	Shera et al. (1995)	[51]	1994	967	59	25	Cross-sectional	Rural	6.101344364	Stratified random sample	NA	NA	NA
Pakistan	Shera et al. (1999)	[52]	1999	1035	73	25	Survey	Rural	7.053140097	Cluster sampling	NA	NA	NA
Pakistan	Shera et al. (2010)	[53]	1998	1852	75	25	Survey	Rural & Urban	4.049676026	Stratified random sample	NA	NA	NA
Pakistan	Mahar et al. (2010)	[54]	2006–2008	19,211	419	30	Cross-Sectional	Rural	2.181042111	Cross-Sectional	NA	NA	NA
Pakistan	Zafer et al. (2011)	[55]	2008	1091	126	30–70	Cross-sectional	Urban	11.54903758	Systematic random	NA	NA	NA
Pakistan	Akhtar et al. (2016)	[56]	2016	1650	132	20–80	Cross-sectional	Rural	8	Cluster sampling	NA	NA	NA
Pakistan	Qureshi et al. (2014)	[57]	2011–2012	815	26	20–70	Survey	Urban	3.190184049	Systematic random	NA	NA	NA
Nepal	Karki (2003)	[58]	1990–1994	1841	86	≥ 20	Survey	Rural & Urban	4.671374253	Cross-sectional	NA	NA	NA
Nepal	Shrestha (2006)	[59]	2001–2002	1012	105	≥ 40	Cross-sectional	Urban	10.37549407	Cluster	NA	NA	NA
Nepal	Mehta (2011)	[60]	2011	736	70	≥ 30	Cross-sectional	Rural & Urban	9.510869565	Simple random	NA	NA	NA
Nepal	Dhungana et al. (2018)	[61]	2014–2015	305	13	18–70	Cross-sectional	Semi-Urban	4.262295082	Simple random	34.4	15.3	17.6
Siri Lanka	Katulanda et al. (2008)	[62]	2005–2006	536	193	≥ 20	Cross-sectional	National	36.00746269	Multi-stage random cluster	NA	NA	NA
Siri Lanka	Fernando (1994)	[63]	1994	33	7	30–64	Cross-sectional	Suburban	21.21212121	Simple random	NA	NA	NA

### Prevalence of undiagnosed-diabetes in South Asian countries

**Fig. 2:**
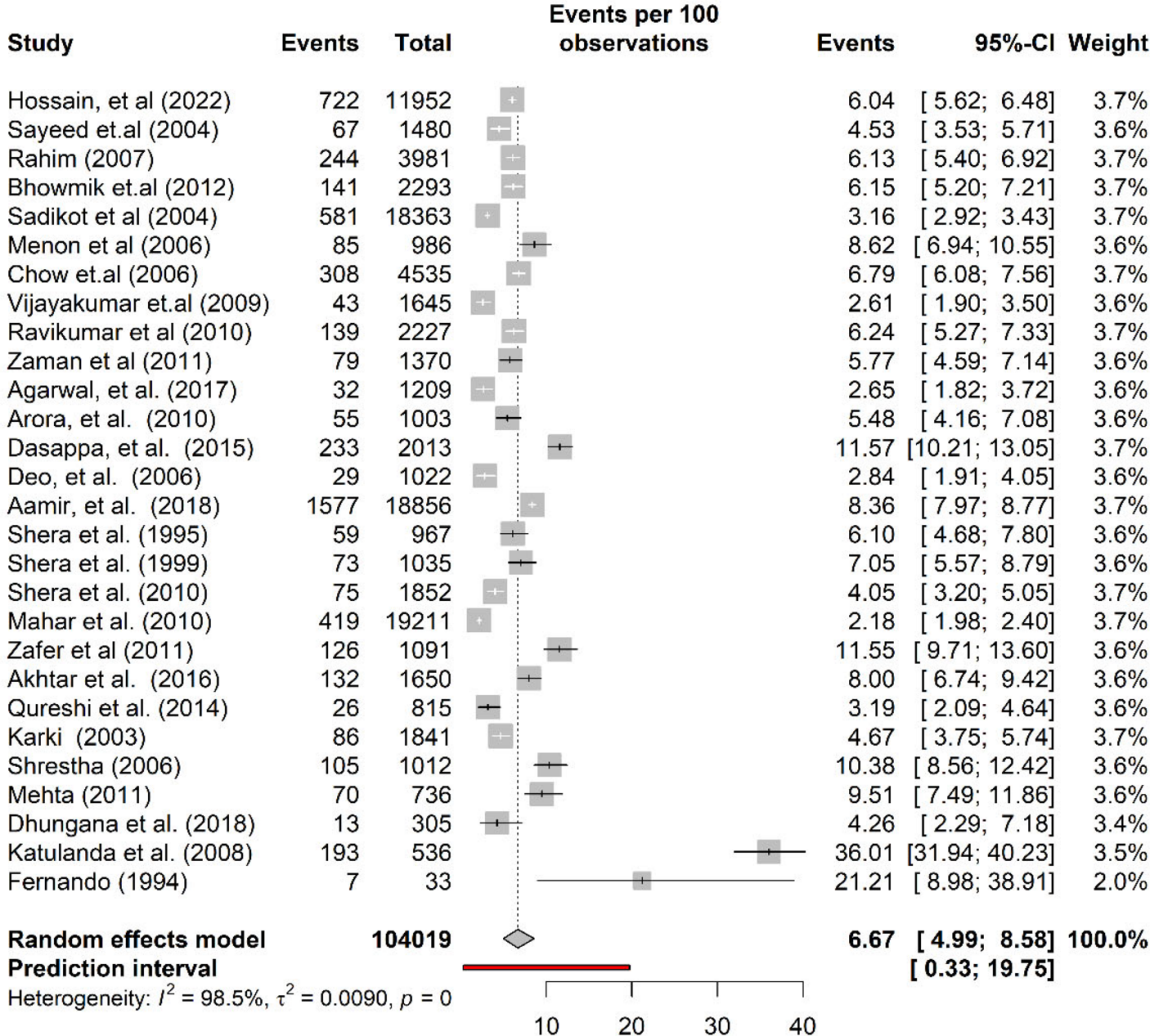
Forest plot of undiagnosed diabetes in South Asian countries

**Fig. 3:**
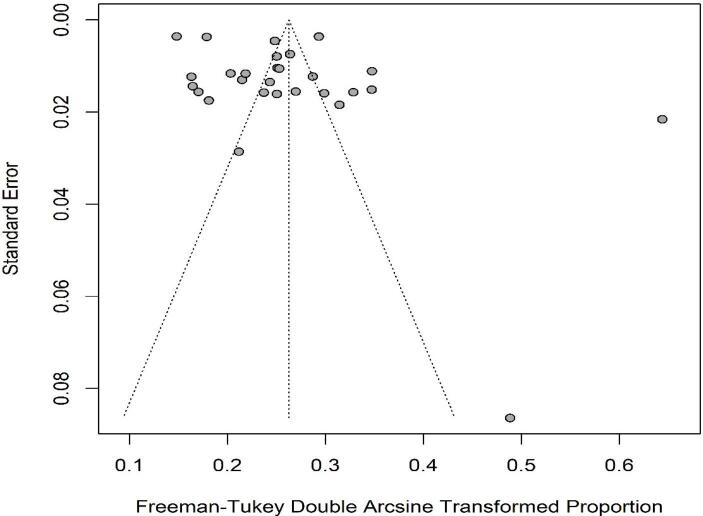
Funnel plot of undiagnosed diabetes in South Asian countries

The pooled prevalence of undiagnosed T2DM across 28 studies (*N* = 104019; events = 5719) was 6.67% (95% CI: 4.99–8.58%); prediction interval: (0.33%–19.75%) (Fig. 2). Substantial heterogeneity was evident (τ² = 0.1007, I² = 98.5%, Q = 1797.38, df = 27, *p* < 0.001), likely due to diagnostic criteria, settings, sampling differences and small sampling size. Funnel plot (Fig. 3) asymmetry suggested potential bias while Egger’s test (t = 1.84, *p* = 0.0773) no publication bias. Small study effect assessment showed asymmetry in the funnel plot. Using the trim and fill method, four studies were found to be potentially missing, bringing the total number of studies to 32 from 28. The adjusted estimated prevalence rate of undiagnosed type 2 diabetes was found to be 4.76% (95% CI: 3.04%–6.82%), as compared to the unadjusted prevalence rate of 6.67%. Between-study heterogeneity persisted even after adjustment (τ² = 0.5332; I² = 98.8%), and the prediction interval was between 1.66% and 25.87%.

Subgroup analyses by country revealed differences in the prevalence of undiagnosed diabetes among the South Asian countries (Table 2, Fig. S1). Sri Lanka had the highest pooled prevalence (30.54%; 95% CI: 17.90–44.82%), followed by Nepal (7.02%; 95% CI: 4.24–10.44%), Pakistan (5.97%; 95% CI: 3.97–8.33%), Bangladesh (5.84%; 95% CI: 5.28–6.42%) and India (5.26%; 95% CI: 3.66–7.14%). Country-specific forest and funnel plots confirmed substantial heterogeneity across most countries (I² ≥ 95%), except for Sri Lanka, where heterogeneity could not be estimated due to a single study.

### Sensitivity analysis

Sensitivity analyses established that no single study had a substantial influence on the pooled prevalence rate of undiagnosed T2DM in South Asia (Fig. S2). The leave-one-out analysis showed that the overall prevalence remained stable after sequential omission of individual studies, indicating the robustness and reliability of the meta-analysis findings.

**Table 2:** Subgroups analysis among the South Asian countries (random effects model)

Country	K	Pooled Prevalence (%)	95% CI	τ²	τ	Q	I² (%)
Bangladesh	4	5.84	5.28–6.42	0.0001	0.0090	10801.66	53.3
India	10	5.26	3.66–7.14	0.0592	0.0613	36547.43	97.5
Pakistan	8	5.97	3.97–8.33	0.0042	0.0650	252.48	99.2
Nepal	4	7.02	4.24–10.44	0.0034	0.0587	19526.74	93.0
Sri Lanka	2	30.54	17.90–44.82	0.0081	0.0898	0.00	67.1

## Discussion

The current systematic review and meta-analysis suggested that the overall prevalence of undiagnosed T2DM among South Asians was 6.67%. These results clearly revealed that there remains a significant percentage of South Asians who have diabetes and do not even know about it. These results also emphasize the fact that due to rapidly increasing urbanization, poor nutrition and diet, lack of exercise, overweight and obesity, and limited access to preventive care in South Asian countries, the prevalence of T2DM among people in this part of the world remains unacceptably high. Even though the prevalence obtained in the current study is relatively low compared to many developing countries [64, 65], this problem cannot be ignored as undiagnosed diabetes may lead to severe complications including cardiovascular diseases, kidney disorders, nerve damage [66], and eye problems.

By addressing this question, the current review offers a comprehensive regional estimate of undiagnosed T2DM prevalence in South Asia. By evaluating 28 studies comprising 104,019 adults from Nepal, Bangladesh, India, Pakistan, and Sri Lanka between 1992 and 2022. Our results showed that the pooled prevalence of undiagnosed T2DM was 6.67% (95% CI: 4.99–8.58%) indicating the meaningful burden of the disease. However, the high heterogeneity (I² = 98.5%) and wide prediction interval (0.33%–19.75%) suggest pronounced variability across settings, populations, study methodologies and small sample size. Overall, these findings have limited generalizability and should not be taken as a precise regional estimate, but rather as a wide indication of variability across studies. These findings should be figured out carefully by focusing on study quality, as variations in quality, study period, and outliers may have participated in the said heterogeneity and altered the pooled estimate. Such heterogeneity reflects differences in methodology of sampling, criteria of diagnosis, characteristics of population and contextual factors such as urban-rural residence, socioeconomic status, and access to health-care facilities. Regarding variation among the countries, Sri Lanka displayed the highest prevalence of undiagnosed T2DM (30.54%); but this result should be deal carefully because it is based on limited data and may reflect contextual factors such as access to health-care facilities and screening attention. On the other hand, India established the lowest prevalence (5.26%). Nepal (7.03%) displayed relatively higher prevalence, which may be associated with urbanization and lifestyle-related risk factors; though, direct comparisons should be treated carefully due to study heterogeneity. Bangladesh (5.84%) and Pakistan (5.97%) demonstrated moderate prevalence levels; yet these estimates are similarly subject to variability in design of study and characteristics of population. Overall, these inter-country differences should be dealt with gently by focusing on the limited number of studies for some countries and the presence of extensive heterogeneity across studies.

Funnel plot asymmetry suggested potential bias and Egger’s test showed no publication bias. While trim-and-fill sensitivity analysis exhibited a pooled prevalence of 4.76%. The full regional generalizability is affected by the lack of data from Bhutana and Maldives, but International Diabetes Federation reported about 9.2% prevalence in Maldives, indicating that the regional burden might be underrated rather than overstated.

Prevalence rates found in this study are lower when compared with those found in many African nations where studies reveal more than 50%-70% diabetes patients have remained undiagnosed till now. Likewise, according to the International Diabetes Federation Diabetes Atlas, the proportion of diabetes patients who go undiagnosed is very high in Sub-Saharan Africa due to inefficient screening programs as well as limited accessibility to health care facilities. On the other hand, in China, prevalence rates for undiagnosed diabetes have been found as high as 8%-12% among the adults in the country [67, 68]. Similarly, in the case of India too, surveys carried out at a national level revealed high prevalence of T2DM patients in urban areas. Again, the prevalence rates of undiagnosed diabetes cases in Middle Eastern countries and Southeast Asian nations have often exceeded 10%, which can be attributed to the rise in the incidence rate of diabetes in developing countries. On the contrary, high-income nations like USA, Canada, and some western European countries have lower prevalence rates of diabetes since they have better healthcare infrastructures as well as efficient screening programs. This inter-country disparity may be due to differences in diagnostic criteria, health care systems, socio-economic environments, lifestyle factors, genetics, and diabetes screening programs. For instance, in South Asia, the prevalence of undiagnosed diabetes may persist because of lack of awareness about diabetes, regular health checks, and differences in access to health care, especially in rural settings. Another reason why a large amount of heterogeneity was found between the included studies is because of the variations in the methods used for diagnosing the condition and the way participants were recruited. Overall, there is a pressing need to develop effective diabetes screening measures for the populations of South Asia in order to combat undiagnosed cases of T2DM.

## Limitations

This study has few limitations that should be considered when interpreting the results. Including data regarding the prevalence of undiagnosed T2DM in this region is limited, not distributed uniformly and some countries (Bhutan, Maldives) do not have valid data, which could affect the overall prevalence. Significant heterogeneity was present in the studies analyzed (I² > 98%) due to variations in terms of sample characteristics, sampling approaches, case definition, geographical locations, and time frames for the research performed in South Asia. While subgroup and sensitivity analysis was carried out, heterogeneity persisted. Furthermore, the pooled estimates relied on crude prevalence estimates from primary sources, while differences in age distribution among samples could impact observed prevalence rates. Additionally, funnel plot asymmetry was identified; nonetheless, any conclusion regarding publication bias, including the use of trim and fill analysis, should be made with caution since extreme heterogeneity can lead to such asymmetry as well. Lastly, most of the studies involved in the analysis had a cross-sectional approach.

## Conclusion

In conclusion, this study found that T2DM in the current region without diagnosis is common among many individuals within this region. Prevalence pooled in the current study could help identify the gaps in the diagnosis of diabetes in this region, while the identified high heterogeneity indicates differences in health-care provision, screening methods, criteria for diagnosis, and social-economic conditions. Differentiations between countries were identified; however, these differences must be considered cautiously due to the small sample size and high heterogeneity, as well as prediction intervals. Small-study effects are evident in the evidence of potential overestimation; sensitivity analysis shows consistency. Hence, these results indicate the importance of developing context-specific and effective screening strategies for the affected population, especially the rural and economically deprived population in the South Asian region.

## Supplementary Information

Below is the link to the electronic supplementary material.

Supplementary Material 1

## Data Availability

No datasets were generated or analysed during the current study.
