# Supplementary material for: Prevalence of undiagnosed type 2 diabetes in South Asia: A systematic review and meta-analysis
Source: Endocrine. 2026 Aug 1;91(1):246. doi: 10.1007/s12020-026-04711-7 (PMC13428718; doi:10.1007/s12020-026-04711-7)
Supplement: Supplementary file 1 — Supplementary Material 1 [file 12020_2026_4711_MOESM1_ESM.docx]

Prevalence of Undiagnosed Type 2 Diabetes in South Asia: A Systematic Review and Meta-Analysis

Faiza Afzal^1^, Imtiaz Ahmad^2*^, Kainat Khalid^3^, Ikram Bashir^4^, Sohail Akhtar^1^

^1^Statistics department, Government College University (GCU), Lahore, Pakistan.

**^2^**Programa de Pós-Graduação Em Bioquímica e Bioprospecção, Universidade Federal de Pelotas, Pelotas, Brasil.

^3^Programa de Pós-Graduação em Enfermagem, Universidade Federal de Santa Maria, Santa Maria, Rio Grande do Sul, Brasil.

**^4^**Laboratório de Botânica, Universidade do Vale do Taquari (Univates), Lajeado, Rio Grande do Sul, Brasil.

**^5^**Department of Mathematics and Statistics, The University of Haripur, Haripur, KP, Pakistan.

^*^Corresponding author: [Imtiazahmad320@gmail.com](mailto:Imtiazahmad320@gmail.com)

0009-0004-1342-7094 [faizaafzalgcu51@gmail.com](mailto:faizaafzalgcu51@gmail.com)

0000-0002-9551-0323 [Imtiazahmad320@gmail.com](mailto:Imtiazahmad320@gmail.com)

0009-0007-5033-6695 [kainat0614@gmail.com](mailto:kainat0614@gmail.com)

0000-0002-0076-4620 [ikrambashir215@gmail.com](mailto:ikrambashir215@gmail.com)

0000-0003-4870-152X [s.akhtar@uoh.edu.pk](mailto:s.akhtar@uoh.edu.pk)

**Table S1** Search Strategies for Electronic Databases.

| **Google Scholar** | ("Diabetes" OR "type 2 diabetes" OR prediabetes OR T2D OR "non-communicable diseases" OR "impaired fasting glucose" OR "impaired glucose tolerance" OR "risk factors" OR "glucose abnormalities" OR "glucose intolerance") AND (Prevalence OR Epidemiology*) AND (Observational* OR "cross-sectional") AND ("Sri Lanka" OR "India" OR "Pakistan" OR "Bangladesh" OR "Nepal" OR "Bhutan" OR "Maldives") |
| --- | --- |
| **PubMed** | ("Diabetes"[Mesh] OR "type 2 diabetes" OR prediabetes OR T2D OR "non-communicable diseases" OR "impaired fasting glucose" OR "impaired glucose tolerance" OR "risk factors" OR "glucose abnormalities" OR "glucose intolerance") AND (Prevalence[Title/Abstract] OR Epidemiology*) AND (Observational Studies [Mesh] OR "cross-sectional") AND ("Sri Lanka" OR "India" OR "Pakistan" OR "Bangladesh" OR "Nepal" OR "Bhutan" OR "Maldives") |
| **EMBASE:** | ('diabetes'/exp OR 'type 2 diabetes' OR prediabetes OR T2D OR 'non-communicable diseases' OR 'impaired fasting glucose' OR 'impaired glucose tolerance' OR 'risk factors' OR 'glucose abnormalities' OR 'glucose intolerance') AND (prevalence OR epidemiology*) AND (observational* OR 'cross-sectional') AND ("Sri Lanka" OR "India" OR "Pakistan" OR "Bangladesh" OR "Nepal" OR "Bhutan" OR "Maldives") |
| Web of Science / ScienceDirect | ("Diabetes" OR "type 2 diabetes" OR prediabetes OR T2D OR "non-communicable diseases" OR "impaired fasting glucose" OR "impaired glucose tolerance" OR "risk factors" OR "glucose abnormalities" OR "glucose intolerance") AND (Prevalence OR Epidemiology*) AND (Observational* OR "cross-sectional") AND ("Sri Lanka" OR "India" OR "Pakistan" OR "Bangladesh" OR "Nepal" OR "Bhutan" OR "Maldives") |

**Table. S1.** Quality assessment table for included studies in the systematic review.

| **S.No** | **Author, year** | **Q1** | **Q2** | **Q3** | **Q4** | **Q5** | **Q6** | **Q7** | **Q8** | **Q9** |
| --- | --- | --- | --- | --- | --- | --- | --- | --- | --- | --- |
| **1** | Hossain et al (2022) | Yes | Yes | Yes | Yes | Yes | Yes | Yes | Yes | Yes |
| **2** | Sayeed et.al (2004) | Yes | Yes | Yes | Yes | No | Yes | Yes | Yes | Yes |
| **3** | Rahim (2007) | Yes | Yes | Yes | Yes | Yes | Unclear | Yes | Yes | Yes |
| **4** | Bhowmik et.al (2012) | Yes | Yes | Yes | Yes | Yes | Yes | Yes | Yes | Yes |
| **5** | Sadikot et al (2004) | Yes | Yes | Yes | Yes | Yes | Unclear | No | Yes | Yes |
| **6** | Menon et al (2006) | Yes | Yes | Yes | Yes | Yes | Yes | Yes | Yes | Yes |
| **7** | Chow CK et.al (2006) | Yes | Yes | Yes | Yes | Yes | Yes | Yes | Yes | Yes |
| **8** | Vijayakumar et.al (2008) | Yes | Yes | Yes | Yes | Yes | Yes | Yes | Yes | No |
| **9** | Ravikumar et al (2010) | Yes | Yes | Yes | Yes | Yes | No | Yes | Yes | Yes |
| **10** | Zaman FA, et al (2011) | Yes | Yes | No | Yes | Yes | Yes | Yes | Yes | Yes |
| **11** | Agarwal, et al. (2017) | Yes | Yes | Yes | Yes | Yes | No | Yes | Yes | Yes |
| **12** | Arora, et al. (2009) | Yes | No | Yes | Yes | Yes | Yes | Yes | Yes | Yes |
| **13** | Dasappa, et al. (2015) | Yes | Yes | Yes | Yes | Yes | Yes | Yes | Yes | Yes |
| **14** | Deo, et al. (2006) | Yes | Yes | Yes | Yes | Yes | Yes | Yes | Yes | Yes |
| **15** | Aamir et al. (2018) | Yes | Yes | Yes | Yes | Yes | Yes | Yes | Yes | Yes |
| **16** | Shera et al. (1995) | Yes | Yes | Yes | Yes | No | Yes | Yes | Yes | Yes |
| **17** | Shera et al. (1999) | Yes | Unclear | No | Yes | Yes | Yes | Yes | Yes | Yes |
| **18** | Shera et al. (2010) | Yes | Yes | Yes | Yes | Yes | Unclear | Yes | Yes | Yes |
| **19** | Mahar et al. (2010) | Yes | Yes | Yes | Yes | Yes | Yes | Yes | Yes | Yes |
| **20** | Zafer et al (2011) | Yes | Yes | Yes | Yes | Yes | Yes | Yes | No | Yes |
| **21** | Akhtar et al. (2016) | Yes | Yes | Yes | Yes | Yes | Yes | Yes | Yes | Yes |
| **22** | Qureshi et al. (2014) | Yes | Yes | Yes | Yes | No | Yes | Yes | Yes | Yes |
| **23** | Karki (2003) | Yes | Yes | No | Yes | Yes | Yes | Yes | Yes | Yes |
| **24** | Shrestha UK (2006) | Yes | Yes | Yes | Yes | Yes | Yes | Yes | Yes | Yes |
| **25** | Mehta KD (2011) | Yes | Yes | Yes | Yes | Yes | No | Yes | No | Yes |
| **26** | Dhungana et al. (2018) | No | Yes | Yes | Yes | Yes | Yes | Yes | Yes | Yes |
| **27** | Katulanda et al. (2008) | Yes | Yes | Yes | Yes | Yes | No | Yes | Yes | Yes |
| **28** | Fernando et al. (1994) | No | Yes | Yes | Yes | Yes | Unclear | Yes | Yes | Yes |

Q1. Was the sample size adequate?

Q2. Were the study subjects and the setting described in detail?

Q3. Was there appropriate statistical analysis?

Q4. Was the sample frame appropriate to address the target population?

Q5. Were study participants sampled in an appropriate way?

Q6. Was the data analysis conducted with sufficient coverage of the identified sample?

Q7. Were valid methods used for the identification of the condition?

Q8. Was the condition measured in a standard, reliable way for all participants?

Q9. Was the response rate adequate, and if not, was the low response rate managed appropriately?


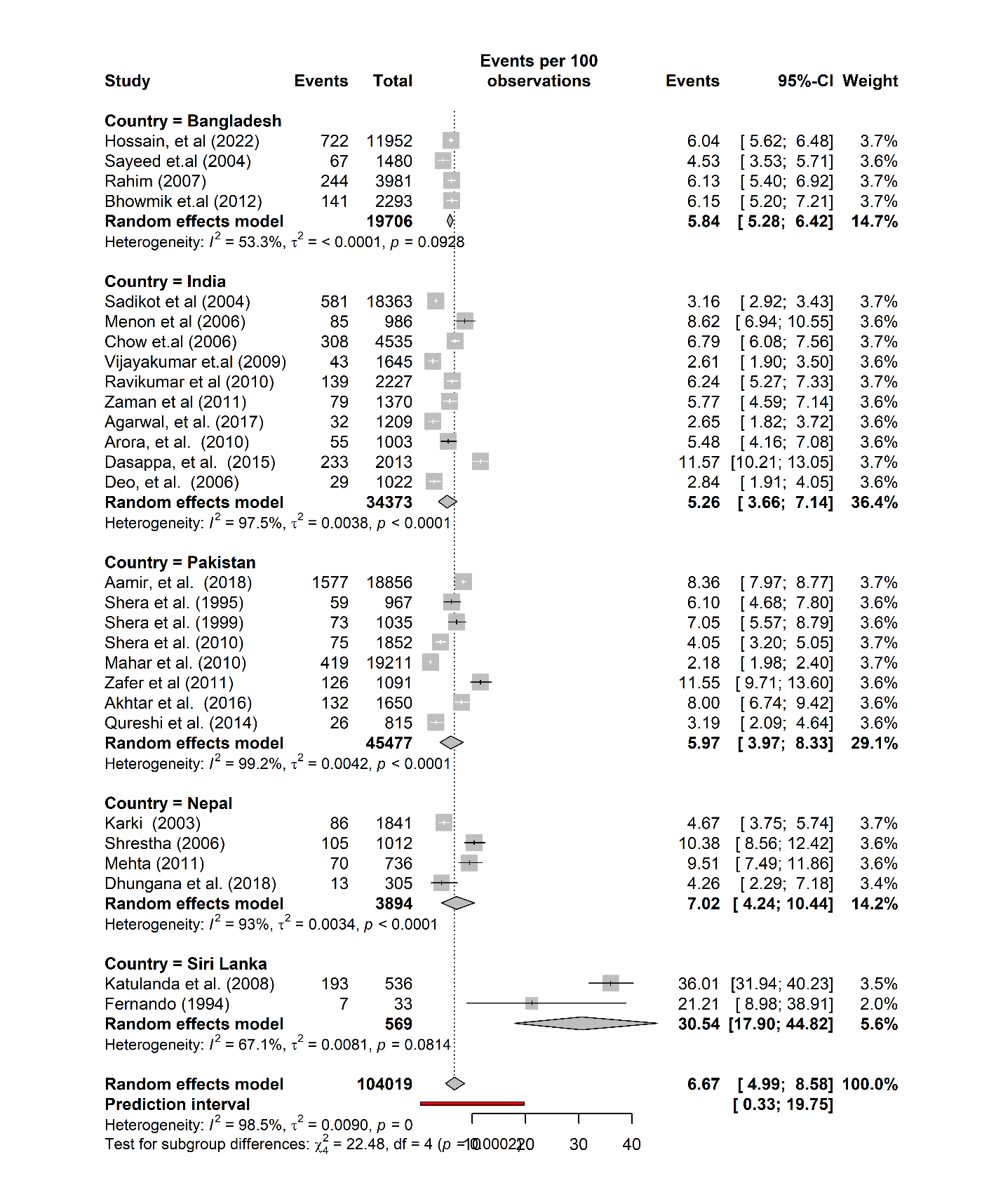


**Figure S1:** Subgroup analysis


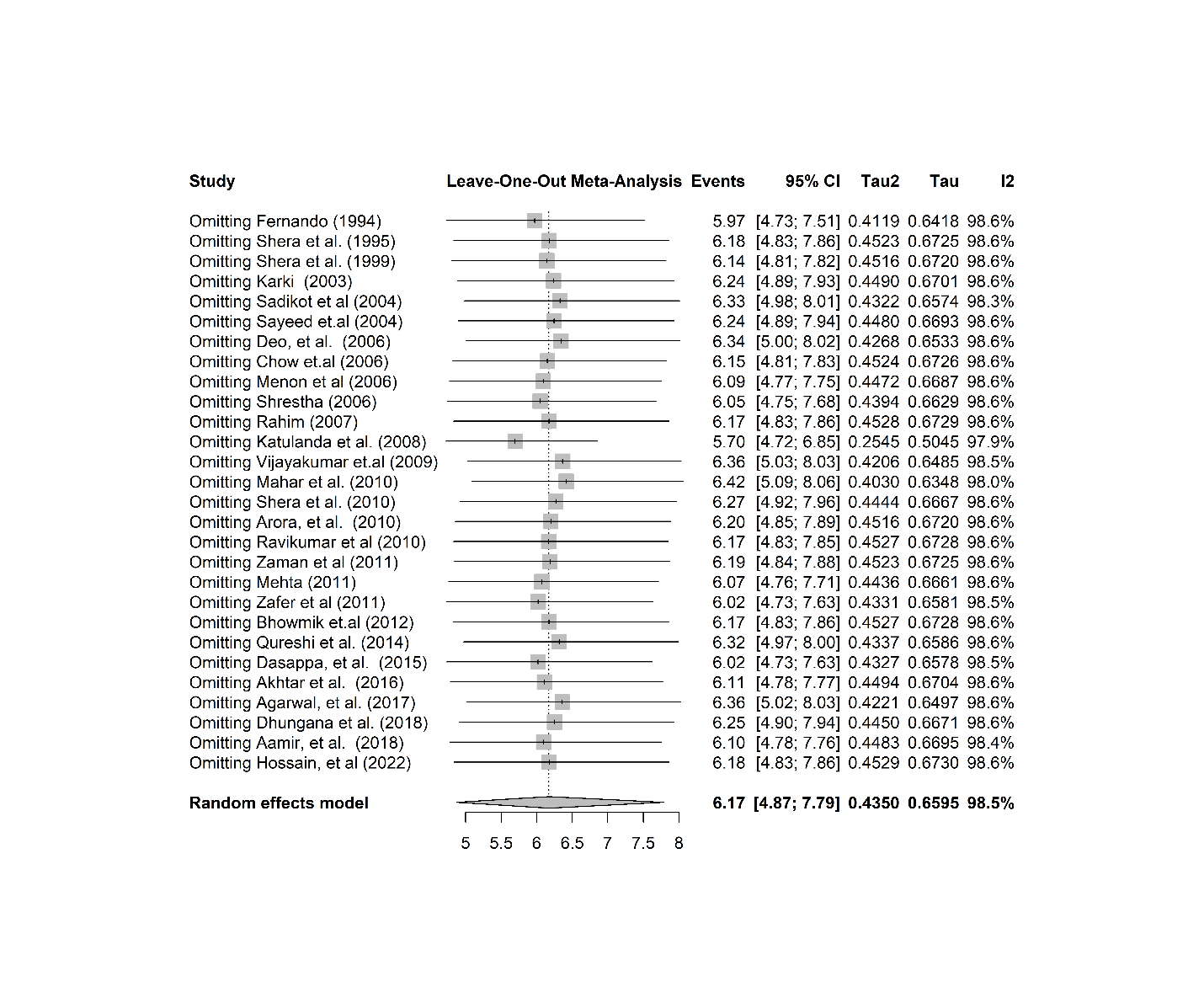


**Figure S2:** Sensitivity Analysis of the selected studies
